# Crystal structure of ethyl (*E*)-4-(4-chlorophen­yl)-4-meth­oxy-2-oxobut-3-enoate

**DOI:** 10.1107/S1600536814017280

**Published:** 2014-08-16

**Authors:** Darlene Correia Flores, Juliano Rosa de Menezes Vicenti, Bruna Ávila Pereira, Gabriele Marques Dias da Silva, Priscilla Jussiane Zambiazi

**Affiliations:** a Escola de Química e Alimentos, Universidade Federal do Rio Grande, Av. Itália km 08, Campus Carreiros, 96203-900 Rio Grande, RS, Brazil; b Departamento de Química, Universidade Federal de Santa Maria, Av. Roraima, Campus, 97105-900, Santa Maria, RS, Brazil

**Keywords:** crystal structure, meth­oxy–ketone inter­actions, benzene–carboxyl­ate carbonyl inter­actions, 4-meth­oxy-2-oxobut-3-enoate ethyl ester

## Abstract

In the title compound, C_13_H_13_ClO_4_, the dihedral angle between the chloro­benezene ring and the least-squares plane through the 4-meth­oxy-2-oxobut-3-enoate ethyl ester residue (r.m.s. deviation = 0.0975 Å) is 54.10 (5)°. In the crystal, mol­ecules are connected by meth­oxy–ketone and benzene–carboxyl­ate carbonyl C—H⋯O inter­actions, generating a supra­molecular layer in the *ac* plane.

## Related literature   

For background to 1,2,4-trielectrophile systems, see: Machado *et al.* (2007 ▶); Siddiqui *et al.* (2013 ▶). For C—H⋯O inter­actions, see: Thakur *et al.* (2010 ▶).
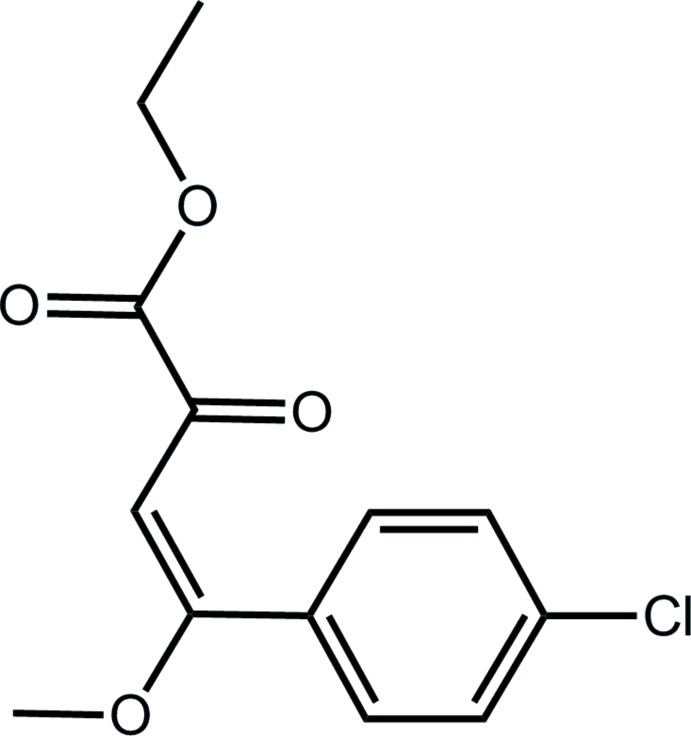


## Experimental   

### 

#### Crystal data  

C_13_H_13_ClO_4_
*M* *_r_* = 268.68
Monoclinic, 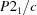

*a* = 9.4557 (4) Å
*b* = 16.6411 (7) Å
*c* = 8.4319 (3) Å
β = 105.644 (2)°
*V* = 1277.64 (9) Å^3^
*Z* = 4
Mo *K*α radiation
μ = 0.30 mm^−1^
*T* = 293 K
0.76 × 0.67 × 0.59 mm

#### Data collection  

Bruker APEXII CCD diffractometer
Absorption correction: gaussian (*XPREP*; Bruker, 2009 ▶) *T*_min_ = 0.667, *T*_max_ = 0.746
30885 measured reflections
3130 independent reflections
2613 reflections with *I* > 2σ(*I*)
*R*_int_ = 0.023

#### Refinement  

*R*[*F* ^2^ > 2σ(*F* ^2^)] = 0.045
*wR*(*F* ^2^) = 0.135
*S* = 1.07
3130 reflections
167 parameters
H atoms treated by a mixture of independent and constrained refinement
Δρ_max_ = 0.40 e Å^−3^
Δρ_min_ = −0.24 e Å^−3^

### 

Data collection: *APEX2* (Bruker, 2009 ▶); cell refinement: *SAINT* (Bruker, 2009 ▶); data reduction: *SAINT*; program(s) used to solve structure: *SHELXS97* (Sheldrick, 2008 ▶); program(s) used to refine structure: *SHELXL97* (Sheldrick, 2008 ▶); molecular graphics: *DIAMOND* (Brandenburg, 2006 ▶); software used to prepare material for publication: *publCIF* (Westrip, 2010 ▶).

## Supplementary Material

Crystal structure: contains datablock(s) I. DOI: 10.1107/S1600536814017280/tk5332sup1.cif

Structure factors: contains datablock(s) I. DOI: 10.1107/S1600536814017280/tk5332Isup2.hkl

Supporting information file. DOI: 10.1107/S1600536814017280/tk5332Isup3.cml

**Fig. 1.**
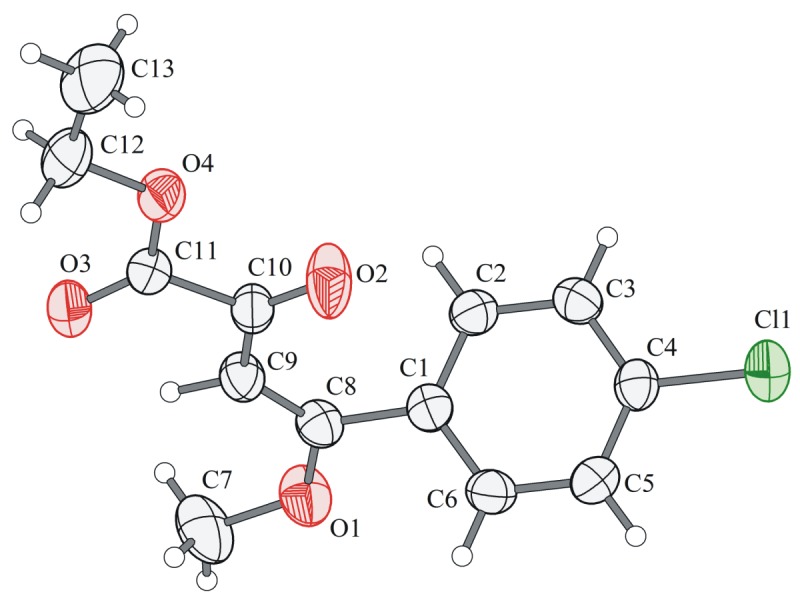
The molecular structure of the title compound showing the atom-labelling scheme and displacement ellipsoids at the 50% probability level.

. DOI: 10.1107/S1600536814017280/tk5332fig1.tif
The mol­ecular structure of the title compound showing the atom-labelling scheme and displacement ellipsoids at the 50% probability level.

**Fig. 2.**
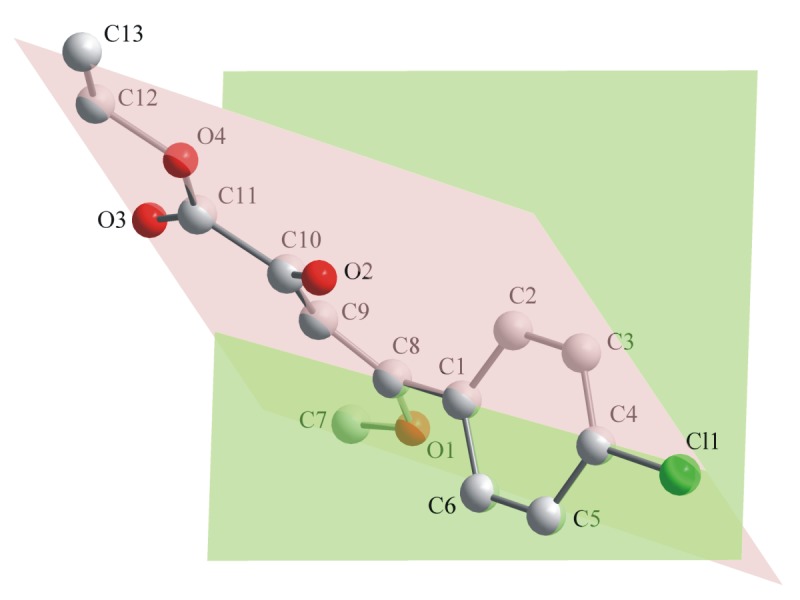
Arrangement between planes within the molecule.

. DOI: 10.1107/S1600536814017280/tk5332fig2.tif
Arrangement between planes within the mol­ecule.

CCDC reference: 1016203

Additional supporting information:  crystallographic information; 3D view; checkCIF report

## Figures and Tables

**Table 1:** Hydrogen-bond geometry (Å, °)

*D*—H⋯*A*	*D*—H	H⋯*A*	*D*⋯*A*	*D*—H⋯*A*
C7—H71⋯O2^i^	0.96	2.54	3.434 (2)	155
C3—H3⋯O3^ii^	0.93	2.60	3.479 (2)	158

Symmetry codes: (i) 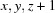; (ii) 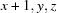.

## supplementary crystallographic information

### S1. Comment

Ethyl-4-aryl-4-methoxy-2-oxo-3-butenoates are interesting precursors for heterocyclic compounds. These 1, 2, 4-trielectrophile systems are synthetic equivalents to 4-aryl-2,4-di,oxobutanoat es (Siddiqui *et al.*, 2013) and were used to produce 1*H*-pyrazoles (Machado *et al.*, 2007). In the title compound (*E*)-Ethyl-4-(4-chlorophenyl)-4-methoxy-2-oxo-3-butenoate, C_13_H_13_O_4_Cl, the whole molecule matches the asymmetric unit (Fig. 1). The molecule presents two almost planar sites (Fig. 2): C7/O1/C8/C9/C10/O2/C11/O3/O4/C12/C13 showed a r.m.s. value of 0.0975 Å with maximum deviation from the mean plane observed for O2 (0.1865 (14) Å). The dihedral angle of 54.10 (5)° confirms that these two fragments are not perfectly perpendicular, suggesting probably the influence of the crystal packing. In the solid state, molecules are connected only through weak non-classical hydrogen bond interactions of the type C—H···O (Thakur *et al.*, 2010), Table 1, generating a supramolecular layer in the *ac* plane.

### S2. Experimental

To a stirred solution of ethyl oxalyl chloride (4.6 ml, 41 mmol) in dry CHCl_3_ (25 ml) at 0 °C, a solution containing the acetal (20 mmol), CHCl_3_ (15 ml) and pyridine (3.25 ml, 41 mmol) were added dropwise. The mixture was left to cool for at least 1 h, then was allowed to warm to room temperature and refluxed for 5 h. The mixture was washed with distilled water (3 times 10 ml) and dried over Na_2_SO_4_. The solvent was evaporated and methyl ethyl oxalate formed was distilled at 80 °C (10 mbar) and solid residue was recrystallized from a diluted solution CHCl_3_. Yield: 14.8 mmol (74%); M.pt: 85–87 °C; ^1^H NMR (400 MHz, CDCl_3_): δ 1.31 (t, 3H, CH_3_), 3.95 (s, 3H, OCH_3_), 4.17 (q, 2H, OCH_2_), 6.28 (s, 1H, C9—H), 7.37 (m, 2H, Ph), 7.43 (m, 2H, Ph); ^13^C NMR (100 MHz, CDCl_3_): δ p.p.m. 13.8 (CH_3_), 57.1 (OCH_3_), 62.1 (OCH_2_), 96.7 (C9), 128.1, 130.5, 132.5, 136.9 (Ph), 163.2 (C11), 174.4 (C8), 180.9 (C10).

### S3. Refinement

With exception of H9 (refined freely), all H atoms attached to C atoms were positioned with idealized geometry (C—H = 0.96 Å for CH_3_, 0.97 Å for CH_2_, and 0.93 Å for aromatic CH) and were refined isotropically with *U*_eq_(H) set to 1.5*U*_eq_(C) for CH_3_ groups, and 1.2 otherwise.

### Crystal data

**Table:**

C_13_H_13_ClO_4_	*F*(000) = 560
*M**_r_* = 268.68	*D*_x_ = 1.397 Mg m^−^^3^
Monoclinic, *P*2_1_/*c*	Melting point: 358 K
Hall symbol: -P 2ybc	Mo *K*α radiation, λ = 0.71073 Å
*a* = 9.4557 (4) Å	Cell parameters from 9103 reflections
*b* = 16.6411 (7) Å	θ = 2.2–28.3°
*c* = 8.4319 (3) Å	µ = 0.30 mm^−^^1^
β = 105.644 (2)°	*T* = 293 K
*V* = 1277.64 (9) Å^3^	Block, yellow
*Z* = 4	0.76 × 0.67 × 0.59 mm

### Data collection

**Table:**

Bruker APEXII CCD diffractometer	3130 independent reflections
Radiation source: fine-focus sealed tube	2613 reflections with *I* > 2σ(*I*)
Graphite monochromator	*R*_int_ = 0.023
φ and ω scans	θ_max_ = 28.3°, θ_min_ = 2.2°
Absorption correction: gaussian (*XPREP*; Bruker, 2009)	*h* = −12→12
*T*_min_ = 0.667, *T*_max_ = 0.746	*k* = −21→22
30885 measured reflections	*l* = −7→11

### Refinement

**Table:**

Refinement on *F*^2^	Primary atom site location: structure-invariant direct methods
Least-squares matrix: full	Secondary atom site location: difference Fourier map
*R*[*F*^2^ > 2σ(*F*^2^)] = 0.045	Hydrogen site location: inferred from neighbouring sites
*wR*(*F*^2^) = 0.135	H atoms treated by a mixture of independent and constrained refinement
*S* = 1.07	*w* = 1/[σ^2^(*F*_o_^2^) + (0.0697*P*)^2^ + 0.4231*P*] where *P* = (*F*_o_^2^ + 2*F*_c_^2^)/3
3130 reflections	(Δ/σ)_max_ < 0.001
167 parameters	Δρ_max_ = 0.40 e Å^−^^3^
0 restraints	Δρ_min_ = −0.24 e Å^−^^3^

### Special details

**Table:**

Experimental. Absorption correction: XPREP (Bruker, 2009) was used to perform the Gaussian absorption correction based on the face-indexed crystal size.
Geometry. All e.s.d.'s (except the e.s.d. in the dihedral angle between two l.s. planes) are estimated using the full covariance matrix. The cell e.s.d.'s are taken into account individually in the estimation of e.s.d.'s in distances, angles and torsion angles; correlations between e.s.d.'s in cell parameters are only used when they are defined by crystal symmetry. An approximate (isotropic) treatment of cell e.s.d.'s is used for estimating e.s.d.'s involving l.s. planes.
Refinement. Refinement of *F*^2^ against ALL reflections. The weighted *R*-factor *wR* and goodness of fit *S* are based on *F*^2^, conventional *R*-factors *R* are based on *F*, with *F* set to zero for negative *F*^2^. The threshold expression of *F*^2^ > σ(*F*^2^) is used only for calculating *R*-factors(gt) *etc*. and is not relevant to the choice of reflections for refinement. *R*-factors based on *F*^2^ are statistically about twice as large as those based on *F*, and *R*- factors based on ALL data will be even larger.

### Fractional atomic coordinates and isotropic or equivalent isotropic displacement parameters (Å^2^)

**Table:**

	*x*	*y*	*z*	*U*_iso_*/*U*_eq_	
Cl1	1.19495 (5)	0.25347 (3)	0.03984 (7)	0.06279 (18)	
O1	0.63288 (14)	0.15936 (9)	0.30504 (14)	0.0569 (3)	
O4	0.32749 (13)	0.05109 (8)	−0.38027 (14)	0.0510 (3)	
O3	0.23337 (14)	0.06369 (8)	−0.16544 (17)	0.0563 (3)	
O2	0.56617 (15)	0.12613 (11)	−0.21718 (15)	0.0712 (5)	
C9	0.50581 (17)	0.11348 (10)	0.03902 (18)	0.0415 (3)	
C11	0.33264 (17)	0.07073 (9)	−0.22767 (19)	0.0399 (3)	
C4	1.02876 (16)	0.22136 (10)	0.06886 (18)	0.0409 (3)	
C8	0.62536 (17)	0.14559 (10)	0.14599 (17)	0.0398 (3)	
C2	0.84722 (17)	0.11991 (9)	0.04448 (19)	0.0409 (3)	
H2	0.8124	0.0685	0.0121	0.049*	
C1	0.76449 (16)	0.17142 (9)	0.11332 (16)	0.0372 (3)	
C3	0.98081 (17)	0.14431 (10)	0.02372 (19)	0.0423 (3)	
H3	1.0373	0.1094	−0.0199	0.051*	
C5	0.94873 (19)	0.27354 (10)	0.1372 (2)	0.0459 (4)	
H5	0.9828	0.3253	0.1666	0.055*	
C6	0.81702 (19)	0.24804 (10)	0.1614 (2)	0.0440 (4)	
H6	0.7633	0.2824	0.2101	0.053*	
C10	0.48443 (16)	0.10629 (10)	−0.13642 (18)	0.0415 (3)	
C12	0.1854 (2)	0.01897 (14)	−0.4754 (2)	0.0622 (5)	
H121	0.1646	−0.0310	−0.4269	0.075*	
H122	0.1078	0.0569	−0.4745	0.075*	
C7	0.5069 (2)	0.14405 (18)	0.3642 (2)	0.0710 (6)	
H71	0.5297	0.1566	0.4794	0.107*	
H73	0.4802	0.0884	0.3478	0.107*	
H72	0.4266	0.1769	0.3049	0.107*	
C13	0.1921 (3)	0.00503 (18)	−0.6446 (3)	0.0811 (7)	
H131	0.0998	−0.0161	−0.7084	0.122*	
H132	0.2689	−0.0327	−0.6444	0.122*	
H133	0.2119	0.0548	−0.6919	0.122*	
H9	0.420 (2)	0.0960 (13)	0.077 (3)	0.056 (5)*	

### Atomic displacement parameters (Å^2^)

**Table:**

	*U*^11^	*U*^22^	*U*^33^	*U*^12^	*U*^13^	*U*^23^
Cl1	0.0423 (3)	0.0725 (3)	0.0767 (3)	−0.0126 (2)	0.0215 (2)	−0.0086 (2)
O1	0.0497 (7)	0.0898 (10)	0.0324 (5)	−0.0089 (7)	0.0130 (5)	−0.0059 (6)
O4	0.0392 (6)	0.0679 (8)	0.0440 (6)	−0.0100 (5)	0.0080 (5)	−0.0148 (5)
O3	0.0433 (6)	0.0666 (8)	0.0639 (7)	−0.0125 (6)	0.0232 (6)	−0.0090 (6)
O2	0.0506 (7)	0.1280 (14)	0.0374 (6)	−0.0351 (8)	0.0159 (5)	−0.0089 (7)
C9	0.0393 (8)	0.0500 (9)	0.0372 (7)	−0.0039 (6)	0.0136 (6)	−0.0013 (6)
C11	0.0365 (7)	0.0392 (7)	0.0438 (7)	−0.0025 (6)	0.0106 (6)	−0.0032 (6)
C4	0.0335 (7)	0.0478 (8)	0.0392 (7)	−0.0022 (6)	0.0060 (5)	0.0011 (6)
C8	0.0406 (8)	0.0463 (8)	0.0331 (7)	0.0010 (6)	0.0109 (6)	0.0009 (6)
C2	0.0446 (8)	0.0370 (7)	0.0413 (7)	−0.0013 (6)	0.0121 (6)	−0.0015 (6)
C1	0.0355 (7)	0.0440 (8)	0.0304 (6)	0.0000 (6)	0.0061 (5)	0.0008 (5)
C3	0.0409 (8)	0.0425 (8)	0.0441 (8)	0.0052 (6)	0.0128 (6)	−0.0016 (6)
C5	0.0443 (8)	0.0431 (8)	0.0491 (8)	−0.0054 (7)	0.0101 (7)	−0.0088 (7)
C6	0.0423 (8)	0.0460 (9)	0.0433 (8)	0.0025 (6)	0.0110 (6)	−0.0090 (6)
C10	0.0361 (7)	0.0510 (9)	0.0387 (7)	−0.0075 (6)	0.0120 (6)	−0.0043 (6)
C12	0.0446 (9)	0.0753 (13)	0.0604 (11)	−0.0159 (9)	0.0032 (8)	−0.0172 (9)
C7	0.0589 (12)	0.120 (2)	0.0396 (9)	−0.0031 (12)	0.0235 (8)	−0.0041 (10)
C13	0.0690 (14)	0.108 (2)	0.0559 (11)	−0.0218 (13)	−0.0014 (10)	−0.0194 (12)

### Geometric parameters (Å, º)

**Table:**

Cl1—C4	1.7386 (16)	C2—H2	0.9300
O1—C8	1.3434 (18)	C1—C6	1.388 (2)
O1—C7	1.432 (2)	C3—H3	0.9300
O4—C11	1.3156 (19)	C5—C6	1.382 (2)
O4—C12	1.4669 (19)	C5—H5	0.9300
O3—C11	1.198 (2)	C6—H6	0.9300
O2—C10	1.2058 (19)	C12—C13	1.463 (3)
C9—C8	1.352 (2)	C12—H121	0.9700
C9—C10	1.443 (2)	C12—H122	0.9700
C9—H9	0.99 (2)	C7—H71	0.9600
C11—C10	1.551 (2)	C7—H73	0.9600
C4—C5	1.376 (2)	C7—H72	0.9600
C4—C3	1.379 (2)	C13—H131	0.9600
C8—C1	1.479 (2)	C13—H132	0.9600
C2—C3	1.382 (2)	C13—H133	0.9600
C2—C1	1.389 (2)		
			
C8—O1—C7	119.46 (14)	C6—C5—H5	120.4
C11—O4—C12	114.34 (13)	C5—C6—C1	120.31 (15)
C8—C9—C10	125.29 (14)	C5—C6—H6	119.8
C8—C9—H9	120.5 (12)	C1—C6—H6	119.8
C10—C9—H9	114.0 (12)	O2—C10—C9	128.48 (15)
O3—C11—O4	125.25 (15)	O2—C10—C11	118.20 (14)
O3—C11—C10	123.32 (14)	C9—C10—C11	113.28 (13)
O4—C11—C10	111.42 (13)	C13—C12—O4	108.48 (17)
C5—C4—C3	121.70 (15)	C13—C12—H121	110.0
C5—C4—Cl1	119.01 (13)	O4—C12—H121	110.0
C3—C4—Cl1	119.30 (13)	C13—C12—H122	110.0
O1—C8—C9	122.91 (14)	O4—C12—H122	110.0
O1—C8—C1	109.03 (12)	H121—C12—H122	108.4
C9—C8—C1	128.07 (13)	O1—C7—H71	109.5
C3—C2—C1	120.57 (14)	O1—C7—H73	109.5
C3—C2—H2	119.7	H71—C7—H73	109.5
C1—C2—H2	119.7	O1—C7—H72	109.5
C6—C1—C2	119.42 (14)	H71—C7—H72	109.5
C6—C1—C8	118.61 (14)	H73—C7—H72	109.5
C2—C1—C8	121.87 (14)	C12—C13—H131	109.5
C4—C3—C2	118.81 (14)	C12—C13—H132	109.5
C4—C3—H3	120.6	H131—C13—H132	109.5
C2—C3—H3	120.6	C12—C13—H133	109.5
C4—C5—C6	119.16 (15)	H131—C13—H133	109.5
C4—C5—H5	120.4	H132—C13—H133	109.5
			
C12—O4—C11—O3	0.5 (2)	C1—C2—C3—C4	−1.6 (2)
C12—O4—C11—C10	−178.46 (15)	C3—C4—C5—C6	0.1 (3)
C7—O1—C8—C9	−3.7 (3)	Cl1—C4—C5—C6	−179.59 (13)
C7—O1—C8—C1	175.94 (18)	C4—C5—C6—C1	−1.7 (3)
C10—C9—C8—O1	172.10 (16)	C2—C1—C6—C5	1.6 (2)
C10—C9—C8—C1	−7.4 (3)	C8—C1—C6—C5	178.17 (15)
C3—C2—C1—C6	0.0 (2)	C8—C9—C10—O2	0.6 (3)
C3—C2—C1—C8	−176.42 (13)	C8—C9—C10—C11	−177.11 (15)
O1—C8—C1—C6	−50.98 (19)	O3—C11—C10—O2	−165.08 (18)
C9—C8—C1—C6	128.62 (18)	O4—C11—C10—O2	13.9 (2)
O1—C8—C1—C2	125.51 (16)	O3—C11—C10—C9	12.9 (2)
C9—C8—C1—C2	−54.9 (2)	O4—C11—C10—C9	−168.14 (14)
C5—C4—C3—C2	1.5 (2)	C11—O4—C12—C13	176.14 (19)
Cl1—C4—C3—C2	−178.80 (12)		

### Hydrogen-bond geometry (Å, º)

**Table:**

*D*—H···*A*	*D*—H	H···*A*	*D*···*A*	*D*—H···*A*
C7—H71···O2^i^	0.96	2.54	3.434 (2)	155
C3—H3···O3^ii^	0.93	2.60	3.479 (2)	158

Symmetry codes: (i) *x*, *y*, *z*+1; (ii) *x*+1, *y*, *z*.
