# Supplementary figures and images for: Crystal structure of ethyl (E)-4-(4-chlorophen­yl)-4-meth­oxy-2-oxobut-3-enoate
Source: Acta Crystallogr Sect E Struct Rep Online. 2014 Aug 16;70(Pt 9):o1025. doi: 10.1107/S1600536814017280 (PMC4186072; doi:10.1107/S1600536814017280)

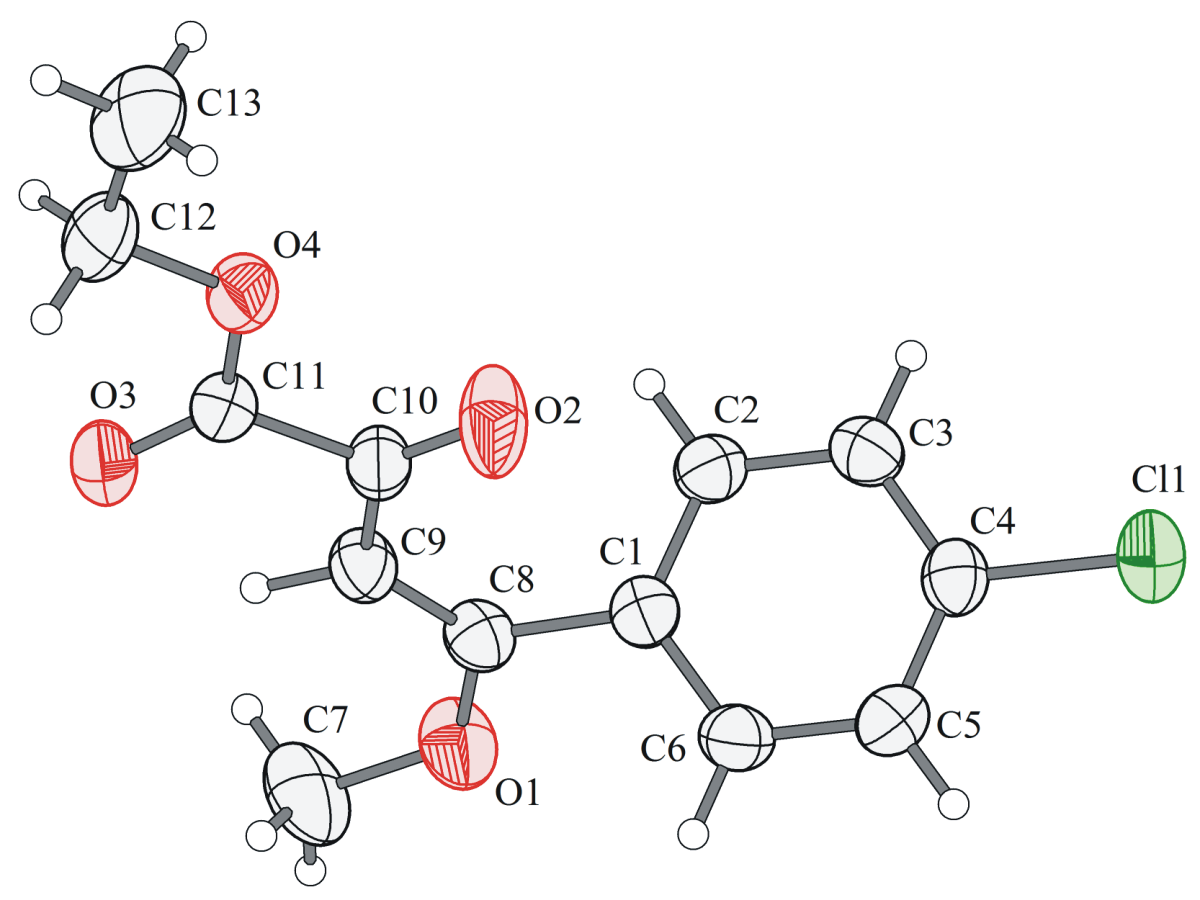

Supplement: Supplementary file 4 [file e-70-o1025-fig1.tif]

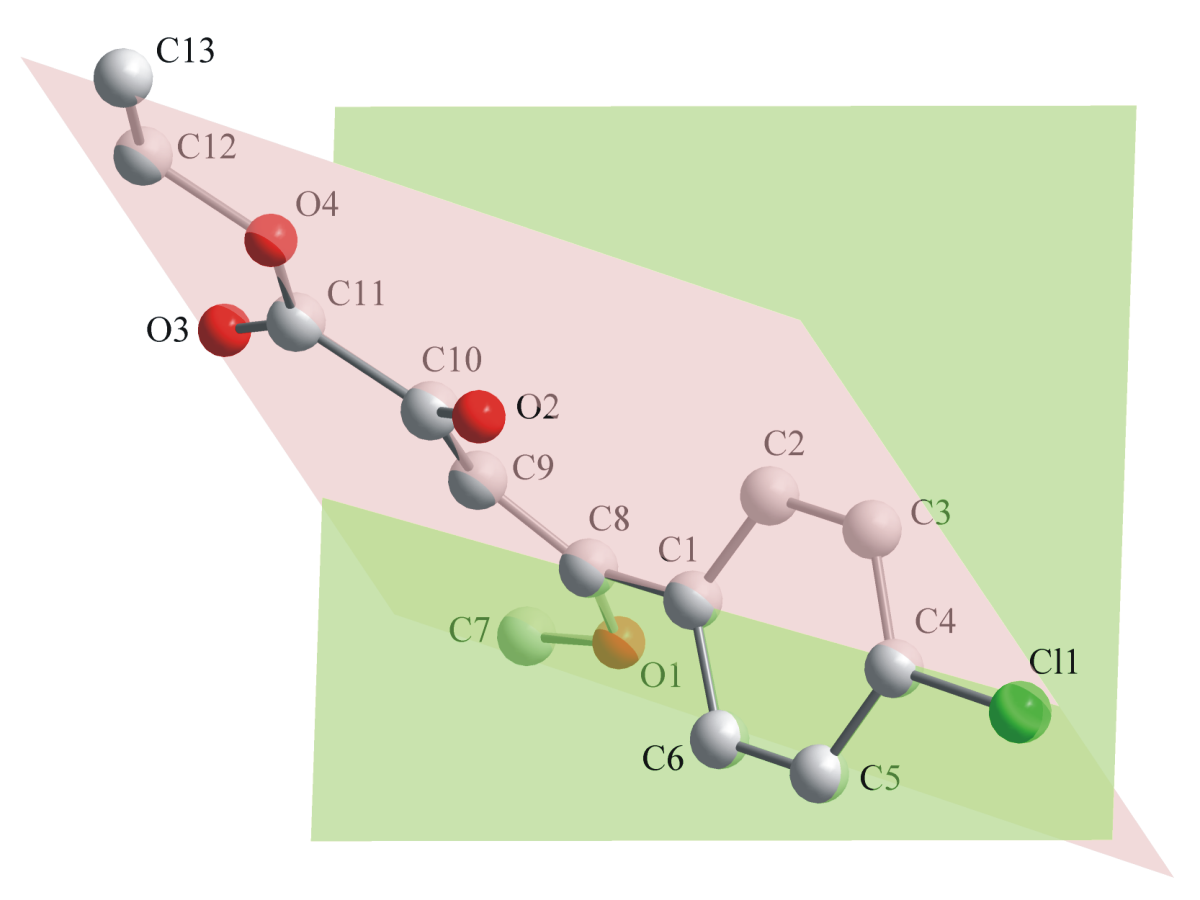

Supplement: Supplementary file 5 [file e-70-o1025-fig2.tif]
